# Exercise training decreases pancreatic fat content and improves beta cell function regardless of baseline glucose tolerance: a randomised controlled trial

**DOI:** 10.1007/s00125-018-4627-x

**Published:** 2018-05-02

**Authors:** Marja A. Heiskanen, Kumail K. Motiani, Andrea Mari, Virva Saunavaara, Jari-Joonas Eskelinen, Kirsi A. Virtanen, Mikko Koivumäki, Eliisa Löyttyniemi, Pirjo Nuutila, Kari K. Kalliokoski, Jarna C. Hannukainen

**Affiliations:** 1 Turku PET Centre, University of Turku, P.O. Box 52, FIN-20521 Turku, Finland; 2 Institute of Neuroscience, National Research Council, Padova, Italy; 3 Turku PET Centre, Turku University Hospital, Turku, Finland; 4 Department of Medical Physics, Turku University Hospital, Turku, Finland; 5 Department of Biostatistics, University of Turku, Turku, Finland; 6 Turku PET Centre, Åbo Akademi University, Turku, Finland

**Keywords:** Beta cell function, Exercise training, High-intensity interval training, Moderate-intensity continuous training, Pancreatic fat content, Pancreatic metabolism, Prediabetes, Type 2 diabetes

## Abstract

**Aims/hypothesis:**

Pancreatic fat accumulation may contribute to the development of beta cell dysfunction. Exercise training improves whole-body insulin sensitivity, but its effects on pancreatic fat content and beta cell dysfunction are unclear. The aim of this parallel-group randomised controlled trial was to evaluate the effects of exercise training on pancreatic fat and beta cell function in healthy and prediabetic or type 2 diabetic participants and to test whether the responses were similar regardless of baseline glucose tolerance.

**Methods:**

Using newspaper announcements, a total of 97 sedentary 40–55-year-old individuals were assessed for eligibility. Prediabetes (impaired fasting glucose and/or impaired glucose tolerance) and type 2 diabetes were defined by ADA criteria. Of the screened candidates, 28 healthy men and 26 prediabetic or type 2 diabetic men and women met the inclusion criteria and were randomised into 2-week-long sprint interval or moderate-intensity continuous training programmes in a 1:1 allocation ratio using random permuted blocks. The primary outcome was pancreatic fat, which was measured by magnetic resonance spectroscopy. As secondary outcomes, beta cell function was studied using variables derived from OGTT, and whole-body insulin sensitivity and pancreatic fatty acid and glucose uptake were measured using positron emission tomography. The measurements were carried out at the Turku PET Centre, Finland. The analyses were based on an intention-to-treat principle. Given the nature of the intervention, blinding was not applicable.

**Results:**

At baseline, the group of prediabetic or type 2 diabetic men had a higher pancreatic fat content and impaired beta cell function compared with the healthy men, while glucose and fatty acid uptake into the pancreas was similar. Exercise training decreased pancreatic fat similarly in healthy (from 4.4% [3.0%, 6.1%] to 3.6% [2.4%, 5.2%] [mean, 95% CI]) and prediabetic or type 2 diabetic men (from 8.7% [6.0%, 11.9%] to 6.7% [4.4%, 9.6%]; *p* = 0.036 for time effect) without any changes in pancreatic substrate uptake (*p* ≥ 0.31 for time effect in both insulin-stimulated glucose and fasting state fatty acid uptake). In prediabetic or type 2 diabetic men and women, both exercise modes similarly improved variables describing beta cell function.

**Conclusions/interpretation:**

Two weeks of exercise training improves beta cell function in prediabetic or type 2 diabetic individuals and decreases pancreatic fat regardless of baseline glucose tolerance. This study shows that short-term training efficiently reduces ectopic fat within the pancreas, and exercise training may therefore reduce the risk of type 2 diabetes.

**Trial registration:**

ClinicalTrials.gov NCT01344928

**Funding:**

This study was funded by the Emil Aaltonen Foundation, the European Foundation for the Study of Diabetes, the Finnish Diabetes Foundation, the Orion Research Foundation, the Academy of Finland (grants 251399, 256470, 281440, and 283319), the Ministry of Education of the State of Finland, the Paavo Nurmi Foundation, the Novo Nordisk Foundation, the Finnish Cultural Foundation, the Hospital District of Southwest Finland, the Turku University Foundation, and the Finnish Medical Foundation.

**Electronic supplementary material:**

The online version of this article (10.1007/s00125-018-4627-x) contains peer-reviewed but unedited supplementary material, which is available to authorised users.

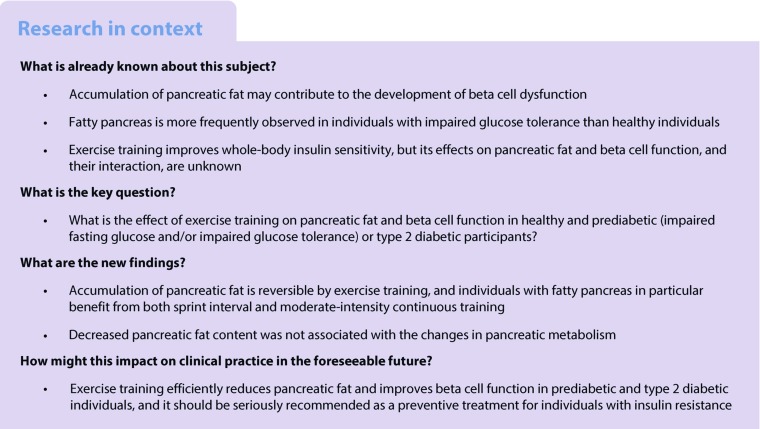



## Introduction

Obesity and physical inactivity are major risk factors for type 2 diabetes mellitus. Obesity has been linked to the accumulation of ectopic fat in different organs, such as the heart, muscle, liver and pancreas [1]. Although ectopic fat in the liver and its association with metabolic disorders has been extensively studied [2], less is known about the role of fatty pancreas despite its clinical significance [3, 4]. A growing amount of evidence suggests that fatty pancreas is more frequently observed in individuals with impaired glucose tolerance [5–9]. Therefore, approaches to maintain a normal pancreatic fat content could reduce the risk of metabolic diseases and type 2 diabetes.

Insulin resistance and dysfunction of the pancreatic beta cells characterise type 2 diabetes and are already present before hyperglycaemia develops [10, 11]. A relationship between pancreatic fat and impaired beta cell function has been shown in some [6, 12] but not all [13–15] studies. A recent study showed that pancreatic fat content decreased after bariatric surgery, with normalisation of the first-phase insulin response, only in individuals with type 2 diabetes despite similar weight losses in type 2 diabetic participants and individuals with normal glucose tolerance, suggesting that fatty pancreas associates with type 2 diabetes [9]. It currently remains unclear whether pancreatic fat accumulation causes beta cell dysfunction and consequently type 2 diabetes, or whether fatty pancreas and type 2 diabetes are independent consequences of obesity [4].

Regular exercise training has a major role in the prevention of type 2 diabetes [16]. It has recently been shown that both moderate-intensity continuous training (MICT) as well as different high-intensity interval training (HIIT) regimes can improve beta cell function in insulin resistance [17–21]. However, the effects of exercise training on pancreatic fat content are unknown, although it has been speculated that lifestyle modifications targeted at decreasing pancreatic fat could improve glycaemic control [22].

To study the effects of short-term exercise training on the pancreas, we recruited healthy middle-aged men as well as men and women with prediabetes or type 2 diabetes. The aims of the present study were to investigate (1) whether 2 weeks of exercise training would have similar effects on pancreatic fat content and beta cell function in healthy and prediabetic or type 2 diabetic men, and (2) whether the effects of sprint interval training (SIT) and MICT would differ in prediabetic or type 2 diabetic men and women. We previously showed that 2 weeks of either SIT or MICT decreased intrathoracic fat in both healthy and prediabetic or type 2 diabetic men [23]. We therefore hypothesised that pancreatic fat would decrease by exercise training similarly in healthy and prediabetic or type 2 diabetic participants.

## Methods

### Study design

This study was a parallel-group randomised controlled trial conducted at Turku PET Centre (Turku, Finland) as a part of a larger study entitled The Effects of Short-time High-intensity Interval Training on Tissue Glucose and Fat Metabolism in Healthy Subjects and Patients With Type 2 Diabetes (NCT01344928). We have previously published several reports of the study focusing on different tissues [23–31]. The first phase of the study investigated healthy men (with measurements between March 2011 and February 2013), and the second phase involved men and women with type 2 diabetes or prediabetes (with measurements between February 2013 and October 2015). The study was conducted according to the Declaration of Helsinki, and the study protocol was approved by the ethical committee of the Hospital District of Southwest Finland, Turku (decision 95/180/2010 §228). The participants’ health status was determined by a thorough physical examination during the screening. The purpose, nature and potential risks of the study were explained verbally and in writing before individuals gave their informed consent to participate in the study.

### Participants

**Fig. 1:**
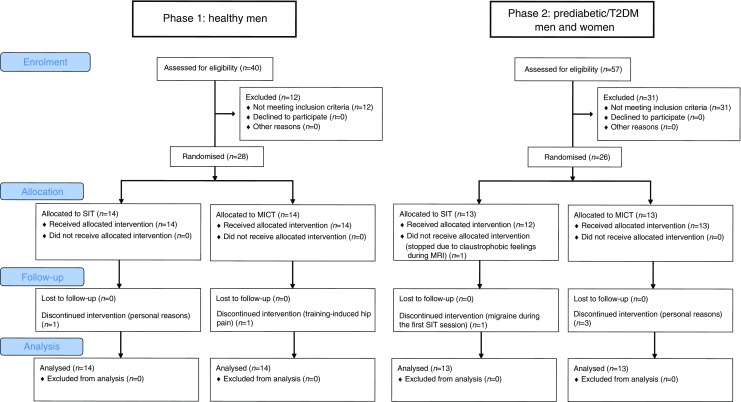
Participant flow diagram. The analyses were carried out using intention-to-treat principle and hence included all the randomised participants. T2DM, type 2 diabetes mellitus

The study was designed to investigate 40–55-year-old participants as type 2 diabetes is often diagnosed within this age range. Individuals with relatively newly diagnosed type 2 diabetes or with prediabetes (impaired fasting glucose and/or impaired glucose tolerance, based on the criteria by ADA) who could benefit from an exercise training intervention were recruited via newspaper announcements. The inclusion criteria for the healthy candidates were: male sex, age 40–55 years, BMI 18.5–30 kg/m^2^, normal glycaemic control verified by OGTT, and no exercise on regular basis ($\overset{\cdot }{V}{\mathrm{O}}_{2\mathrm{peak}}$ ≤ 40 ml kg^−1^ min^−1^). For prediabetic or type 2 diabetic candidates, the inclusion criteria were the same, except: male or female sex, BMI 18.5–35 kg/m^2^, and impaired glucose tolerance or type 2 diabetes according to ADA criteria [32]. A candidate was excluded if he or she had a condition which could potentially endanger their health during the study or interfere with the interpretation of the results as explained in detail previously [26, 31]. Of 97 screened individuals, 28 healthy men and 26 prediabetic or type 2 diabetic individuals (16 men, ten women) fulfilled the inclusion criteria and were admitted into the study (Fig. 1). Of 26 prediabetic or type 2 diabetic individuals, the ADA criteria for type 2 diabetes [32] were met in 17 (11 men); 13 (ten men) of these were being treated with at least one type of oral hypoglycaemic agent (median duration of type 2 diabetes 4 years), whereas four individuals with type 2 diabetes (one man) had taken no previous medication for type 2 diabetes. The remaining nine prediabetic or type 2 diabetic participants (five men) met the ADA criteria for prediabetes, having impaired fasting glucose and/or impaired glucose tolerance [32].

Randomisation to the SIT and MICT groups using a 1:1 allocation ratio was performed separately for the healthy and prediabetic or type 2 diabetic participants with random permuted blocks, as previously described in detail [23, 27, 28]. Given the nature of the intervention, no blinding was used. Two healthy and five prediabetic or type 2 diabetic participants discontinued the trial (see Fig. 1 for details).

### Training interventions

Training interventions consisted of six exercise sessions over 2 weeks [25, 31]. SIT consisted of 4–6 episodes of all-out cycling effort (Monark Ergomedic 894E; Monark, Vansbro, Sweden) lasting 30 s each, with a supramaximal workload, separated by 4 min of recovery. The MICT group cycled (Tunturi E85; Tunturi Fitness, Almere, the Netherlands) for 40–60 min at an intensity equalling 60% of peak workload. Training interventions are described in detail in electronic supplementary material (ESM Methods).

### Outcome measures

The number of completed experiments in terms of outcome measures is summarised in Table 1. The reasons for not completing the experiments have previously been explained in detail [27, 28].

**Table 1:** Number of completed experiments in the study

Variable	Healthy men		Prediabetic or type 2 diabetic participants			
	Pre	Post	Pre		Post	
			Men	Women	Men	Women
Participants with at least one measurement	28	26	16	10	13	8
Pancreatic fat content, completed	23	21	12	8	12	6
Pancreatic glucose uptake, completed	22	23	14	10	12	7
Pancreatic fatty acid uptake, completed	23	17	16	8	12	5
Beta cell function, completed	28	23	16	10	13	8

#### Pancreatic fat content

The primary outcome measure of the study was pancreatic fat content, which was determined by proton magnetic resonance spectroscopy (^1^H MRS) using a Philips Gyroscan Intera 1.5 T CV Nova Dual Scanner (Philips Medical Systems, Best, the Netherlands) with a SENSE body coil (Philips Medical Systems, Best, the Netherlands). Details of the protocol are described in ESM Methods.

#### Pancreatic metabolism

As secondary outcomes, pancreatic glucose and fatty acid uptake were studied by positron emission tomography (PET) after an overnight fast. Fatty acid uptake was studied in the fasted state using 14(*R*,*S*)-[^18^F]fluoro-6-thia-heptadecanoic acid ([^18^F]FTHA; 155 (SD 9) MBq) as a tracer. On a different day, glucose uptake was measured using 2-deoxy-2-[^18^F]fluoro-d-glucose ([^18^F]FDG; 157 (SD 10) MBq) during a hyperinsulinaemic–euglycaemic clamp when participants had reached a stable glucose concentration of 5.0 (±0.5) mmol/l [23, 26]. Details of PET image processing and analysis are described in ESM Methods.

#### Beta cell function variables, glycaemic control, and anthropometrics

Measurement of whole-body insulin-stimulated glucose uptake (*M* value), details of OGTT, and determination of body composition and peak exercise capacity ($\overset{\cdot }{V}{\mathrm{O}}_{2\mathrm{peak}}$) are described in ESM Methods. Insulin secretion rates (ISRs) were calculated from C-peptide deconvolution for every 5 min for the whole 2 h period of the OGTT [33]. Early- and late-phase ISR (ISR_early_ and ISR_late_) were calculated as the AUC of ISR from 0 to 30 min and from 30 to 120 min, respectively. Total ISR (ISR_total_) denotes the AUC for the whole 2 h period. An index of early ISR normalised to glucose concentration (ΔISR_0-30_/ΔG_0-30_) was calculated as (ISR_30_-ISR_0_)/(glucose_30_-glucose_0_). Other beta cell function variables were derived by modelling as described by Mari et al [34] and described in detail in ESM Methods.

### Statistical analysis

Sample size was calculated for the whole study (NCT01344928) based on its primary outcome, skeletal muscle glucose uptake [25, 29]. No sample size calculation was performed specifically on the outcome measures of the present study.

The normal distribution of the variables was tested using the Shapiro–Wilk test and evaluated visually. Logarithmic (log_10_) or square root transformations were performed when appropriate to achieve a normal distribution. Statistical analyses were performed using hierarchical mixed linear models with compound symmetry covariance structure. First, the differences between healthy and prediabetic or type 2 diabetic men were studied with the model, which included one within-factor term (time; indicating the overall mean change between baseline and measurement after the intervention), one between-factor term (diabetes mellitus; healthy and prediabetic or type 2 diabetic men) and one interaction term (time × diabetes mellitus; indicating whether mean change during the study was different between healthy and prediabetic or type 2 diabetic men). Prediabetic and type 2 diabetic women were completely excluded when comparing the effects of exercise in healthy and prediabetic or type 2 diabetic participants to avoid mixing the effects of sex and glucose intolerance. Second, differences between SIT and MICT in prediabetic or type 2 diabetic participants, including both men and women (reported in ESM Methods), were studied using a model that included within-factor time, between-factor group (SIT and MICT) and interaction terms (time × group; whether the mean change was different in the SIT and MICT groups). The analyses were carried out using the intention-to-treat principle and included all the randomised participants. Missing data points were accounted for by restricted maximum likelihood estimation within the linear mixed models. Correlations were calculated using Pearson’s correlation (Spearman’s rank correlation for non-normally distributed data). The statistical tests were performed as two-sided and the level of statistical significance was set at 0.05. The analyses were performed using SAS System, version 9.4 for Windows (SAS Institute, Cary, NC, USA).

## Results

### Healthy vs prediabetic or type 2 diabetic men

The effects of exercise training were first studied separately in prediabetic and type 2 diabetic men (ESM Table 1). As most of the variables changed similarly in these groups, prediabetic and type 2 diabetic men were combined into one group. Therefore, the effects of exercise training are compared between healthy and prediabetic or type 2 diabetic men.

Prediabetic or type 2 diabetic men were heavier, had more fat and had a lower exercise capacity than healthy men at baseline (Table 2). Exercise training improved $\overset{\cdot }{V}{\mathrm{O}}_{2\mathrm{peak}}$ and *M* value similarly in the healthy and prediabetic or type 2 diabetic men, and gave rise to a small but statistically significant decrease in waist circumference, fat percentage, subcutaneous and visceral fat, and HbA_1c_ in both groups (Table 2).

**Table 2:** Participant characteristics of healthy and prediabetic or type 2 diabetic men and glycaemic control

Variable	Healthy men		Men with prediabetes or T2DM		*p* value		
	Pre (*n* = 28)	Post (*n* = 26)	Pre (*n* = 16)	Post (*n* = 13)	Baseline difference	Time	Time × DM
Prediabetic/T2DM participants (*n*)	–	–	5/11	4/9			
Age (years)	48 (46, 50)		49 (48, 51)		0.14		
Weight (kg)	83.6 (79.7, 87.5)	83.3 (79.4, 87.2)	96.3 (91.2, 101.5)	96.2 (91.0, 101.3)	<0.001*	0.20	0.80
BMI (kg/m^2^)	26.1 (25.1, 27.1)	26.0 (25.0, 27.0)	30.4 (29.1, 31.8)	30.4 (29.0, 31.7)	<0.001*	0.17	0.70
Waist circumference (cm)	95.5 (92.4, 98.6)	94.8 (91.7, 98.0)	105.3 (101.0, 109.6)	104.7 (100.4, 109.0)	<0.001*	0.018*	0.84
Fat (%)	22.6 (20.9, 24.3)	21.7 (20.0, 23.3)	28.8 (26.5, 31.2)	28.1 (25.7, 30.4)	<0.001*	<0.001*	0.78
Subcutaneous fat (kg)^a^	4.09 (3.69, 4.53)	4.04 (3.64, 4.04)	5.58 (4.87, 6.41)	5.52 (4.87, 6.41)	<0.001*	0.030*	0.93
Visceral fat (kg)^a^	3.05 (2.70, 3.44)	2.98 (2.64, 3.36)	4.22 (4.97, 3.59)	4.08 (4.80, 3.47)	0.002*	0.002*	0.54
$\overset{\cdot }{V}{\mathrm{O}}_{2\mathrm{peak}}$ (ml kg^−1^ min^−1^)	34.2 (32.7, 35.7)	35.7 (34.2, 37.2)	29.3 (27.2, 31.4)	30.0 (27.9, 32.1)	<0.001*	0.003*	0.23
*M* value (μmol kg^−1^ min^−1^)	35.3 (30.0, 40.6)	38.7 (33.3, 44.1)	17.5 (10.3, 24.8))	21.6 (14.2, 29.0)	<0.001*	0.007*	0.80
HbA_1c_ (mmol/mol)	36.9 (35.2, 38.6)	34.8 (33.0, 36.5)	39.6 (37.3, 41.8)	37.5 (35.2, 39.9)	0.071	<0.001*	0.87
HbA_1c_ (%)	5.5 (5.4, 5.7)	5.3 (5.2, 5.5)	5.8 (5.6, 6.0)	5.6 (5.4, 5.8)	0.080	<0.001*	0.90
Fasting glucose (mmol/l)^b^	5.5 (5.3, 5.7)	5.7 (5.5, 6.0)	7.2 (6.9, 7.6)	7.1 (6.8, 7.5)	<0.001*	0.26	0.086
Fasting insulin (pmol/l)^b^	4.8 (3.9, 6.0)	6.0 (4.7, 7.5)	14.5 (10.9, 19.3)	13.6 (10.0, 18.5)	<0.001*	0.37	0.11
Fasting NEFA (mmol/l)	0.70 (0.62, 0.77)	0.62 (0.54, 0.70)	0.69 (0.60, 0.78)	0.68 (0.58, 0.78)	0.86	0.072	0.15
OGTT 2 h glucose (mmol/l)	5.8 (5.0, 6.6)	6.0 (5.1, 6.8)	11.2 (10.1, 12.2)	10.3 (9.2, 11.4)	<0.001*	0.16	0.058
OGTT 2 h insulin (pmol/l)^b^	26.8 (21.2, 33.9)	27.3 (21.2, 35.1)	66.9 (49.4, 90.7)	64.4 (46.0, 90.0)	<0.001*	0.93	0.82
OGTT glucose AUC (mmol/l × min)	845 (774, 916)	887 (812, 961)	1342 (1250, 1435)	1323 (1225, 1421)	<0.001*	0.67	0.25

Results are mean (95% CI) for age. For all other variables, the results are model-based means (95% CI)

The baseline difference *p* value indicates whether there is a baseline difference between healthy and prediabetic or type 2 diabetic men. The time *p* value displays the mean change between pre- and post-measurements. The Time × DM *p* value indicates whether the mean changes are different between healthy and prediabetic or type 2 diabetic men

^a^Square root transformation performed

^b^Logarithmic transformation (log_10_) performed

*p ≤ 0.05

DM, diabetes mellitus; T2DM, type 2 diabetes mellitus

**Fig. 2:**
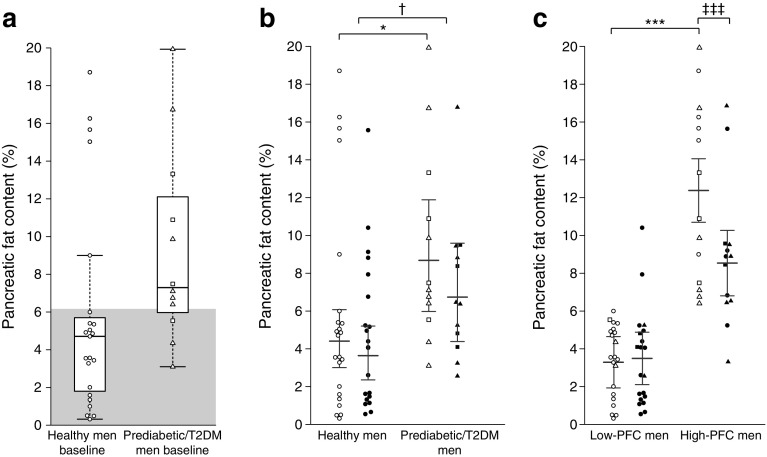
Pancreatic fat content in healthy and prediabetic or type 2 diabetic men at baseline (**a**), before and after the training intervention when participants were grouped into healthy and prediabetic or type 2 diabetic men (**b**), and before and after the training intervention when men were grouped according to low (≤6.2%) and high (>6.2%) pancreatic fat at baseline (**c**). The shaded area in (**a**) denotes normal pancreatic fat content (≤6.2%). (**b**, **c**) Square root transformation was performed to calculate model-based means and 95% CI. Circles, healthy men; squares, prediabetic men; triangles, type 2 diabetic men; white symbols, before exercise intervention; black symbols, after exercise intervention. T2DM, type 2 diabetes mellitus. *p ≤ 0.05, ***p ≤ 0.001 for baseline difference between the groups; ^†^*p* ≤ 0.05 for time effect; ^‡‡‡^*p* ≤ 0.001 time effect for men with high pancreatic fat content

Pancreatic fat content was lower in healthy men than prediabetic or type 2 diabetic men at baseline (*p* = 0.032; Fig. 2a,b). Two weeks of exercise training decreased pancreatic fat similarly in the healthy (from 4.4% [3.0%, 6.1%] to 3.6% [2.4%, 5.2%]) and prediabetic or type 2 diabetic men (from 8.7% [6.0%, 11.9%] to 6.7% [4.4%, 9.6%], *p* = 0.036 for time, *p* = 0.52 for the interaction time × diabetes mellitus; Fig. 2b). Five healthy men had pancreatic fat content greater than 6.2%, which has been recommended as the cut-off value for normal pancreatic fat [22], whereas three prediabetic or type 2 diabetic men had pancreatic fat below 6.2% (Fig. 2a). When the men were divided into groups with low (below 6.2%) and high (above 6.2%) pancreatic fat content, exercise training decreased pancreatic fat by 31% only in those men who had fatty pancreas at baseline (*p* = 0.001 for the interaction time × pancreatic fat content; *p* < 0.001 for the time effect in men with high pancreatic fat) (Fig. 2c). In the men’s pooled baseline data, pancreatic fat correlated positively with BMI, fat percentage, visceral fat and fasting glucose concentration (Table 3).

**Table 3:** Correlations between pancreatic fat content and whole-body and beta cell variables in all men

Variable	Pancreatic fat content (%)			
	Baseline, all men		Changes, all men	
	*r*	*p*	*r*	*p*
BMI (kg/m^2^)	0.42	0.012*	0.28	0.30
Fat (%)	0.45	0.007*	0.05	0.81
Visceral fat (kg)	0.59	<0.001*	0.19	0.33
*M* value (μmol kg^−1^ min^−1^)	−0.28	0.12	−0.20	0.36
HbA_1c_ (mmol/mol)	0.18	0.30	−0.30	0.14
Fasting glucose (mmol/l)	0.35	0.040*	−0.11	0.60
Fasting insulin (pmol/l)	0.28	0.10	−0.11	0.59
Fasting NEFA (mmol/l)	−0.28	0.13	−0.08	0.71
Pancreatic glucose uptake (μmol 100 g^−1^ min^−1^)	−0.12	0.55	0.23	0.28
Pancreatic fatty acid uptake (μmol 100 g^−1^ min^−1^)	−0.18	0.33	−0.02	0.93
ISR_basal_ (pmol min^−1^ m^−2^)	0.41	0.015*	−0.06	0.77
ISR_early_ (nmol/m^2^)	0.18	0.30	−0.21	0.32
ISR_total_ (nmol/m^2^)	0.42	0.014*	−0.10	0.63
Glucose sensitivity (pmol min^−1^ m^−2^ [mmol/l]^−1^)	−0.14	0.41	−0.02	0.92
Rate sensitivity (pmol m^−2^ [mmol/l]^−1^)	0.06	0.75	−0.05	0.82
Potentiation factor ratio	−0.26	0.14	0.10	0.62

*Statistically significant *p* value (*p* ≤ 0.05)

Pancreatic fatty acid uptake and insulin-stimulated glucose uptake determined by PET were similar in the healthy and prediabetic or type 2 diabetic men at baseline, and remained unchanged after 2 weeks of exercise training (Table 4).

**Table 4:** Pancreatic metabolism and beta cell function in healthy and prediabetic or type 2 diabetic men

Variable	Healthy men		Prediabetic/T2DM men		*p* values		
	Pre (*n* = 28)	Post (*n* = 26)	Pre (*n* = 16)	Post (*n* = 13)	Baseline difference	Time	Time × DM
Pancreatic metabolism							
Glucose uptake (μmol 100 g^−1^ min^−1^)	3.9 (3.6, 4.2)	4.0 (3.7, 4.3)	3.7 (3.3, 4.1)	3.8 (3.4, 4.3)	0.53	0.31	0.97
Fatty acid uptake (μmol 100 g^−1^ min^−1^)^a^	1.4 (1.2, 1.6)	1.2 (1.0, 1.5)	1.3 (1.0, 1.5)	1.2 (1.0, 1.5)	0.46	0.38	0.54
Beta cell function							
ISR_basal_ (pmol min^−1^ m^−2^)	81 (69, 94)	89 (76, 102)	152 (136, 169)	139 (122, 157)	<0.001*	0.54	0.006*
ISR_early_ (nmol m^−2^)^b^	7.5 (6.4, 8.7)	9.1 (7.7, 10.7)	9.1 (7.4, 11.1)	8.5 (6.9. 10.6)	0.15	0.23	0.028*
ΔISR_0–30_/ΔG_0–30_ (nmol m^−2^/mmol l^−1^)^b^	0.16 (0.13, 0.19)	0.12 (0.10, 0.15)	0.08 (0.06, 0.10)	0.07 (0.05, 0.09)	<0.001*	0.010*	0.71
ISR_late_ (nmol/m^2^)	32 (28, 36)	32 (28, 36)	41 (36, 46)	41 (36, 47)	0.005*	0.98	0.85
ISR_total_ (nmol/m^2^)	40 (36, 45)	42 (37, 46)	50 (45, 56)	50 (44, 56)	0.008*	0.75	0.65
Glucose sensitivity (pmol min^−1^ m^−2^ [mmol/l]^−1^)	114 (94, 133)	114 (94, 133)	61 (35, 86)	58 (31, 84)	0.001*	0.81	0.81
Rate sensitivity (pmol m^−2^ [mmol/l]^−1^)	1043 (836, 1250)	842 (620, 1065)	726 (453, 1000)	452 (156, 748)	0.12	0.013*	0.69
Potentiation factor ratio^a^	2.0 (1.7, 2.4)	1.9 (1.6, 2.3)	1.3 (1.0, 1.7)	1.7 (1.3, 2.2)	0.010*	0.29	0.086

Results are presented as model-based means (95% CI)

The baseline difference *p* value indicates whether there is a baseline difference between healthy and prediabetic or type 2 diabetic men. The Time *p* value displays the mean change between pre- and post-measurements. The Time × DM *p* value indicates whether the mean changes are different between healthy and prediabetic or type 2 diabetic men

^a^Square root transformation performed

^b^Logarithmic transformation (log_10_) performed

*Statistically significant *p* value (*p* ≤ 0.05)

DM, diabetes mellitus; T2DM, type 2 diabetes mellitus

**Fig. 3:**
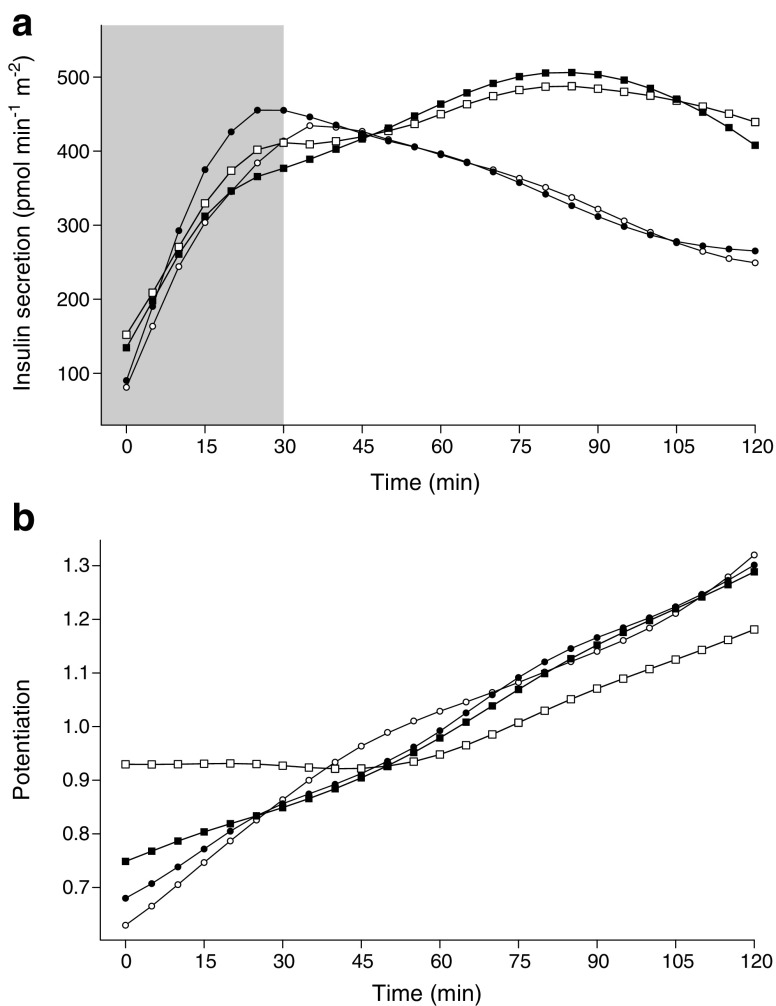
ISR (**a**) and potentiation (**b**) during 2 h OGTTs in healthy and prediabetic or type 2 diabetic men before and after the training intervention. The shaded area in (**a**) denotes ISR_early_ (0–30 min), which increased only in healthy men (*p* = 0.006 for the time effect in healthy men). There were non-significant differences in the potentiation of insulin secretion between prediabetic or type 2 diabetic men and healthy men (*p* = 0.083 for time effect for the potentiation factor ratio in prediabetic or type 2 diabetic men). White circles, healthy men before the exercise intervention; black circles, healthy men after the exercise intervention; white squares, prediabetic or type 2 diabetic men before the exercise intervention; black squares, prediabetic or type 2 diabetic men after the exercise intervention

ISR_basal_, ISR_late_ and ISR_total_ were higher in prediabetic or type 2 diabetic men than healthy men at baseline, while ISR_early_ did not differ between the groups (Table 4, Fig. 3a). Exercise training decreased ISR_basal_ in prediabetic or type 2 diabetic men (*p* = 0.034 for the time effect in prediabetic or type 2 diabetic men), and increased ISR_early_ only in the healthy men (*p* = 0.006 for the time effect in healthy men; Fig. 3a). However, the index of early ISR normalised for glucose concentration (ΔISR_0-30_/ΔG_0-30_) decreased similarly in both groups (*p* = 0.010 for time). Before the intervention, ISR_basal_ and ISR_total_ correlated positively with pancreatic fat content in the whole study population, but no correlations were found between changes in pancreatic fat and beta cell function (Table 3).

At baseline, the potentiation factor ratio was lower in prediabetic or type 2 diabetic men than healthy men (p = 0.010). Two weeks of exercise training had a non-significantly different effect on potentiation in healthy and prediabetic or type 2 diabetic men (*p* = 0.086 for the interaction time × diabetes mellitus; Fig. 3b).

Pancreatic glucose sensitivity was lower in prediabetic or type 2 diabetic men than healthy men at baseline, and remained unchanged by training (Table 4). Rate sensitivity was not statistically significantly different at baseline, and it decreased similarly after training in both healthy and prediabetic or type 2 diabetic men (Table 4).

### SIT vs MICT in prediabetic or type 2 diabetic men and women

The effects of exercise training did not differ between men and women (ESM Table 2), and therefore the effects of SIT and MICT were studied in the combined group of men and women with prediabetes or type 2 diabetes. As previously reported [25], SIT and MICT decreased fat percentage, abdominal fat and HbA_1c_ to a similar extent, and increased *M* value, in prediabetic or type 2 diabetic participants (ESM Table 3). However, $\overset{\cdot }{V}{\mathrm{O}}_{2\mathrm{peak}}$ improved only after SIT (ESM Table 3) [25]. Both training modes decreased pancreatic fat content in those individuals with fatty pancreas at baseline (*p* = 0.035 for time, *p* = 0.47 for the interaction time × group). The decrease in ISR_basal_ and ISR_early_ was not significant (*p* = 0.082 and *p* = 0.056 for time, respectively), and ΔISR_0-30_/ΔG_0-30_ decreased (*p* = 0.005 for time; ESM Table 4), after training. ISR_late_ and ISR_total_ remained unchanged. The potentiation factor ratio increased (*p* = 0.030 for time) and rate sensitivity decreased (*p* = 0.007 for time). Except for $\overset{\cdot }{V}{\mathrm{O}}_{2\mathrm{peak}}$, there was no difference between SIT and MICT in prediabetic or type 2 diabetic participants (ESM Tables 3 and 4). Baseline pancreatic fat content did not correlate with any of the whole-body or beta cell function variables in prediabetic or type 2 diabetic men and women (ESM Table 5).

## Discussion

The present study shows for the first time that exercise training decreases pancreatic fat content regardless of baseline glucose tolerance. Both SIT and MICT reduced pancreatic fat, especially in individuals with fatty pancreas, underlining the beneficial effect of exercise training for those at risk of type 2 diabetes. Decreased pancreatic fat was not associated with changes in pancreatic metabolism or beta cell function.

At baseline, pancreatic fat content was higher in prediabetic or type 2 diabetic men than healthy men, which is consistent with previous studies [5–8], although a conflicting report also exists [35]. Whereas pancreatic fat was positively associated with BMI, body fat and visceral fat in all male participants, these associations were lost when considering only those who had prediabetes or type 2 diabetes. Furthermore, pancreatic fat correlated positively with fasting glucose, ISR_basal_ and ISR_total_ in all men, but not in prediabetic or type 2 diabetic participants. Previous studies have reported conflicting results with regards to the association between pancreatic fat and BMI, some reporting a positive correlation [5, 7, 12, 35] and others reporting no significant correlation [6, 22]. The association between pancreatic fat and beta cell function is equally unclear. Studies addressing mainly non-diabetic individuals have reported no association between these variables [13–15], whereas other studies have shown that the association is different in normoglycaemic and prediabetic or type 2 diabetic individuals [6, 12]. Beta cell functional variables have been shown to have distinct patterns of decrease when spanning the range from normoglycaemic obese individuals to those with overt type 2 diabetes [36], and even beta cell defects in impaired fasting glucose and impaired glucose tolerance are different [37]. Therefore, it may be that different factors affect pancreatic fat accumulation during normoglycaemia, impaired glucose tolerance and full-blown type 2 diabetes [6, 9, 22]. These discrepancies highlight the fact that more research is needed to better understand the causes and consequences of fatty pancreas.

Just 2 weeks of exercise training decreased pancreatic fat similarly in healthy and prediabetic or type 2 diabetic men. A cross-sectional study investigating eight monozygotic young adult male twin pairs with different fitness levels reported no difference in pancreatic fat between more and less active twins [38]. However, even the healthy participants in the present study had relatively low physical fitness and high BMI, which may explain why such a short training intervention decreased pancreatic fat in the present study. Pancreatic fatty acid uptake and insulin-stimulated glucose uptake as well as fasting serum NEFA concentration were similar in healthy and prediabetic or type 2 diabetic men at baseline and remained unchanged by training. Hence, substrate uptake does not seem to explain the baseline difference between the groups or the observed decrease in pancreatic fat after exercise training. However, we measured fatty acid uptake in the fasting state, and it is possible that fat accumulation may occur during the postprandial period. A subgroup comparison between prediabetic and type 2 diabetic men (ESM Table 1) suggests that glucose uptake may be different during the progression of type 2 diabetes. Moreover, sex may also affect pancreatic metabolism (ESM Table 2). However, the small number of participants in the subgroup comparisons limits the interpretation of the findings. Further studies spanning the range from obesity to overt type 2 diabetes could shed more light on the question of whether there is a distinct pattern in pancreatic metabolism when type 2 diabetes progresses, and whether it is related to the accumulation of pancreatic fat.

When dividing the men according to low (≤6.2%) and high (>6.2%) baseline pancreatic fat content [22], exercise training decreased pancreatic fat by 31% in those men who had fatty pancreas to start with. The result that as little as 2 weeks of exercise has a marked impact on those individuals with fatty pancreas is clinically significant, as ectopic fat accumulation is recognised as a major factor in the development of type 2 diabetes [3, 4, 22].

The effects of exercise training on beta cell function have been previously studied in obese, prediabetic or type 2 diabetic individuals using a disposition index as the measure of beta cell function. Regardless of the different exercise modes (HIIT, MICT or functional high intensity training [CrossFit]) used in different studies, all have reported an increased disposition index after the training intervention, and hence inferred that training improves beta cell function [17–21]. However, as the disposition index may be biased [39], we studied beta cell function using several model-based variables. At baseline, ISR_total_ was higher in prediabetic or type 2 diabetic men than healthy men. This reflects higher glucose levels and the reciprocal relationship between insulin sensitivity and insulin secretion, implying that reduced insulin sensitivity is compensated by increased ISR [40] until glucotoxicity becomes too great for beta cells to compensate sufficiently [41]. After exercise training, ISR_basal_ decreased in prediabetic or type 2 diabetic men, while ISR_early_ increased only in healthy men. When considering all prediabetic or type 2 diabetic participants (men and women), differences in these variables after SIT and MICT were not significant (*p* = 0.082 and *p* = 0.056 for time, respectively). On the other hand, whole-body insulin sensitivity increased similarly in both groups. The increase in ISR_early_ in healthy men may be a response to improved glucose sensitivity in the muscles, whereas prediabetic or type 2 diabetic individuals may compensate improved whole-body insulin sensitivity by maintaining or decreasing insulin secretion, which was already increased at baseline. A similar compensatory decrease in insulin secretion in overweight adults after training has previously been reported [21]. When normalising early ISR for glucose concentration, it decreased similarly in healthy and prediabetic or type 2 diabetic men. A corresponding decrease was observed in rate sensitivity, probably due to improved whole-body insulin sensitivity.

The potentiation of insulin secretion was impaired in prediabetic or type 2 diabetic men compared with healthy men at baseline. Our finding is in line with previous studies, which have reported blunted and delayed potentiation in diabetic individuals using a multiple meal test [42] as well as a decreased potentiation factor ratio in diabetic individuals compared with non-diabetic control participants [6]. Exercise training might normalise potentiation in prediabetic or type 2 diabetic men towards that of the healthy men (Fig. 3b; *p* = 0.083), suggesting that exercise training may improve the ability of beta cells to read potentiating signals, such as incretins and neural signals. However, further work is necessary to explore this.

Over the past few years, numerous studies have elucidated the effects of HIIT, or its special case SIT, in both healthy and type 2 diabetic participants, and have concluded that HIIT is at least as beneficial as the more traditional MICT in improving glycaemic control and maximal exercise capacity [43–45]. With regards to prediabetic or type 2 diabetic men and women in the present study, SIT and MICT had a different effect only on $\overset{\cdot }{V}{\mathrm{O}}_{2\mathrm{peak}}$, which increased only after SIT, as discussed in our previous report [25]. The changes observed in all the other variables investigated in the present study, including increased whole-body insulin sensitivity, decreased pancreatic fat content, improved potentiation and decreased ΔISR_0-30_/ΔG_0-30_, were similar for both training modes. To conclude, both SIT and MICT can be used to improve the metabolic health of prediabetic or type 2 diabetic individuals.

The present study is not, however, without limitations. The number of participants was relatively small, although similar sample sizes have previously been used in exercise training studies with a technically demanding study design. In addition, the dropout rate was relatively high. The prediabetic or type 2 diabetic participants comprised a rather heterogeneous group containing both men and women, some with prediabetes and others with type 2 diabetes, but the number of each was too small to fully address the differences between the subgroups. It has been shown that pancreatic function differs during prediabetes and overt type 2 diabetes [36]. However, in the prediabetic and type 2 diabetic men in the present study, pancreatic function and responses to exercise were quite similar, probably because the individuals with type 2 diabetes had been relatively recently diagnosed (median duration of type 2 diabetes 4 years). Also, the oral hypoglycaemic medication taken by the participants with type 2 diabetes was interrupted for 2 days before the pre- and post-measurement PET scans. However, measuring glucose and fatty acid uptake as well as pancreatic fat content were unsuccessful in some participants (Table 1), and the heterogeneity of the prediabetic or type 2 diabetic individuals may have affected the results relating to pancreatic metabolism.

Using ^1^H MRS to measure pancreatic fat content cannot distinguish intracellular fat accumulation in beta cells from adipose tissue infiltration. Since pancreatic islets containing beta cells cover only around 2% of the pancreatic mass, most of the fat detected by MRS probably lies outside the islets. While the main deposition of fat in the human pancreas remains unclear, it has been suggested that ^1^H MRS measurement of triacylglycerols in the whole pancreas represents a surrogate marker for islet lipids [3]. In addition, individuals with type 2 diabetes have been shown to have a lower pancreatic volume than healthy individuals [46], making voxel placement more challenging. In the present study, the voxel placement within the body of pancreas was carefully ensured by axial, sagittal and coronal directions of investigation.

Finally, this study was designed to investigate the early-phase responses to exercise training. Lim et al studied the effects of dietary energy restriction in type 2 diabetes at different time points over 8 weeks, showing that although liver fat content decreased rapidly, the decrease in pancreatic fat content and improvement in beta cell function took longer to occur [47]. Therefore, the lack of association between changes in pancreatic fat content and beta cell function in the present study may be due to the short time course of the exercise intervention, and longer exercise interventions will be needed to investigate the functional effect of decreased pancreatic fat.

## Conclusion

This study shows for the first time that exercise training decreases pancreatic fat content regardless of baseline glucose tolerance. In particular, individuals with fatty pancreas benefited from exercise training, with a similar decrease obtained with both SIT and MICT. As an accumulation of ectopic fat in the internal organs, including the pancreas, is a key factor in obesity and the development of type 2 diabetes, this study shows that exercise training is an effective way to decrease ectopic fat accumulation and hence reduce the risk of type 2 diabetes.

## Electronic supplementary material

ESM (PDF 145 kb)

## Data Availability

The data are available on reasonable request from the corresponding author.

## Abbreviations

HIIT: High-intensity interval training
ISR: Insulin secretion rate
MICT: Moderate-intensity continuous training
MRS: Magnetic resonance spectroscopy
*M* value: Whole-body insulin-stimulated glucose uptake
PET: Positron emission tomography
SIT: Sprint interval training
