# Supplementary material for: Exercise training decreases pancreatic fat content and improves beta cell function regardless of baseline glucose tolerance: a randomised controlled trial
Source: Diabetologia. 2018 May 2;61(8):1817–28. doi: 10.1007/s00125-018-4627-x (PMC6061150; doi:10.1007/s00125-018-4627-x)
Supplement: Supplementary file 1 — (PDF 145 kb) [file 125_2018_4627_MOESM1_ESM.pdf]

ELECTRONIC SUPPLEMENTARY MATERIAL

Heiskanen *et al*: Exercise training decreases pancreatic fat content and improves beta cell function regardless of baseline glucose tolerance: A randomised controlled trial

Supplementary materials and methods

Training interventions

The training interventions consisted of six exercise sessions within two weeks [1, 2]. Each session was performed in supervised laboratory conditions. The SIT training consisted of 4-6 x 30 s of all-out cycling efforts with 4 min of recovery (Monark Ergomedic 894E, Monark, Vansbro, Sweden). The number of bouts was increased from 4 to 5, and further to 6 after every other session. The participants were familiarized with SIT during the screening phase (2 x 30 s bouts). Each bout started with 5-second acceleration to maximal cadence without any resistance, followed by a sudden increase of the load (7.5% of whole-body weight in kg for healthy men, 10% of fat free mass in kg for prediabetic or type 2 diabetic participants) and maximal cycling for 30 s. The MICT group cycled (Tunturi E85, Tunturi Fitness, Almere, The Netherlands) for 40-60 min at the intensity of 60% of peak workload. The duration of cycling was increased from 40 min to 50 min, and further to 60 min after every other session.

Outcome measures

Pancreatic fat content. The primary outcome of the study was pancreatic fat content, which was determined by proton magnetic resonance spectroscopy (^{1}H MRS) with Philips Gyroscan Intera 1.5 T CV Nova Dual Scanner (Philips Medical Systems, Best, the Netherlands) with a SENSE body coil (Philips Medical Systems, Best, the Netherlands). Volume of interest (12 mm x 10 mm x 15 mm) was placed on body of pancreas using axial, sagittal, and coronal images. Molecular contents of lipids and water were determined using single-voxel proton spectroscopy with PRESS sequence using TE of 27

msec and TR of 3000 msec. A total of 10 spectra was collected as a time series, each with 4 measurements. Imaging was done in breath hold. Measurement was performed twice for each patient, once with water suppression and once without it. Data was analyzed using the linear combination of model spectra software package (LCModel) version 6.3-0C with the LCMgui [3]. Lipid ratio (peaks at 0.9 ppm, 1.3 ppm, and 1.6 ppm) to water was measured. Fat and water amplitudes were corrected for different T2 decay, and molar concentrations of ^{1}H nuclei in fat and water were corrected as described before [4].

Pancreatic metabolism. As secondary outcomes, pancreatic glucose and fatty acid uptake were studied by positron emission tomography (PET) after overnight fast. The PET imaging was performed with GE Advance PET/CT scanner (General Electric Medical System, Milwaukee, WI, USA). Fatty acid uptake was studied at fasted state using 14(*R*,*S*)-[^{18}F] fluoro-6-thia-heptadecanoic acid ([^{18}F]FTHA; 155 (SD 9) MBq) as a tracer. On a separate day, glucose uptake was measured using 2-deoxy-2-[^{18}F]fluoro-D-glucose ([^{18}F]FDG; 157 (SD 10) MBq) during a euglycemic hyperinsulinemic clamp (see below) when the participants had reached the stable glucose concentration of 5.0 (±0.5) mmol·l^{-1}. PET image raw files were corrected for attenuation, dead time, and decay. Images were reconstructed using 3D-OSEM procedure and analyzed using Carimas software (version 2.7, www.turkupetcentre.fi/carimas). Three-dimensional volumes of interests (VOIs) were drawn on the tail, body, and head of pancreas. Average of all three VOIs was taken to obtain a tissue time activity curve. Graphical analysis was used to quantify the fractional uptake rate. Glucose and fatty acid uptake rates were calculated by multiplying corresponding fractional uptake rate values with mean plasma glucose or NEFA concentrations during the imaging period, respectively. Pancreatic lump constant was set at 1 [5].

Glucose clamp and OGTT. Whole-body insulin-stimulated glucose uptake rate (*M* value), the gold-standard measure of insulin sensitivity in humans [6], was measured during euglycemic hyperinsulinemic clamp as described in detail previously [7, 8]. An OGTT was performed in the

morning after at least 10 h of fasting. Liquid containing 75 g of glucose (Nutrical; Nutricia Medical, Turku, Finland) was given, and blood samples were taken at the baseline and at 15, 30, 60, 90, 120 min during the test to determine glucose, C-peptide, and insulin concentrations [8].

Beta cell function parameters. Beta cell function was described by various parameters. Insulin secretion rates (ISR) were calculated from C-peptide deconvolution for every 5 min for the whole 2-h period of OGTT [9]. Early- and late-phase ISR were calculated as the area under curve (AUC) of ISR from 0 to 30 min and from 30 to 120 min, denoted by ISR_{early} and ISR_{late}, respectively. Total ISR (ISR_{total}) denotes AUC during the whole 2-h period. An index of early ISR normalized to glucose concentration ($\Delta ISR_{0-30}/\Delta G_{0-30}$) was calculated as ($ISR_{30}$-$ISR_{0}$)/(glucose$_{30}$-glucose$_{0}$). Other parameters were derived by modeling as described by Mari et al [10]. Glucose sensitivity was defined as the mean slope of the glucose-insulin secretion dose-response curve, and it describes the degree of pancreatic beta cell responsiveness to absolute blood glucose levels. Potentiation factor includes various potentiating signals (glucose-induced potentiation, secretory influence of gastrointestinal hormones, neural factors) and it was calculated every 5 min for 2-h period. Potentiation is reported as potentiation factor ratio, calculated as mean(potentiation$_{110-120}$)/mean(potentiation$_{0-10}$). Finally, rate sensitivity represents the dependence of insulin secretion on the rate of change in plasma glucose concentration, accounting for anticipation of insulin secretion as glucose levels rise.

Body composition and peak exercise capacity. VO_{2peak} was determined by a maximal exercise test on a cycle ergometer (Ergoline 800 s; VIASYS Healthcare, Germany) at Paavo Nurmi Center (Turku, Finland) as described previously [1, 2, 7]. Body composition was measured by the bioimpedance monitor (InBody 720, Mega Electronics Ltd., Kuopio, Finland).

References

1. Sjöros T, Heiskanen MA, Motiani KK, et al (2018) Increased insulin-stimulated glucose uptake in both leg and arm muscles after sprint interval and moderate intensity training in subjects with Type 2 Diabetes or Prediabetes. Scand J Med Sci Sport 28:77–87

2. Kiviniemi AM, Tulppo MP, Eskelinen JJ, et al (2014) Cardiac autonomic function and high-

intensity interval training in middle-age men. Med Sci Sports Exerc 46:1960–1967

3. Provencher SW (1993) Estimation of metabolite concentrations from localized in vivo proton NMR spectra. Magn Reson Med 30:672–9

4. Hannukainen JC, Borra R, Linderborg K, et al (2011) Liver and pancreatic fat content and metabolism in healthy monozygotic twins with discordant physical activity. J Hepatol 54:545–552

5. Peltoniemi P, Lönnroth P, Laine H, et al (2000) Lumped constant for [(18)F]fluorodeoxyglucose in skeletal muscles of obese and nonobese humans. Am J Physiol Endocrinol Metab 279:E1122–30

6. DeFronzo RA, Tobin JD, Andres R (1979) Glucose clamp technique: a method for quantifying insulin secretion and resistance. Am J Physiol 237:E214–E223

7. Heiskanen MA, Leskinen T, Eskelinen J-J, et al (2015) Different Predictors of Right and Left Ventricular Metabolism in Healthy Middle-Aged Men. Front Physiol 6:389

8. Honkala SM, Motiani KK, Eskelinen J-J, et al (2017) Exercise training reduces intrathoracic fat regardless of defective glucose tolerance. Med Sci Sport Exerc 49:1313–22

9. Van Cauter E, Mestrez F, Sturis J, Polonsky KS (1992) Estimation of insulin secretion rates from C-peptide levels: Comparison of individual and standard kinetic parameters for C-peptide clearance. Diabetes 41:368–377

10. Mari A, Schmitz O, Gastaldelli A, et al (2002) Meal and oral glucose tests for assessment of beta-cell function: modeling analysis in normal subjects. Am J Physiol Endocrinol Metab 283:E1159–E1166

ESM Table 1. Effects of exercise training on glycemic control, pancreatic metabolism, and beta-cell function in prediabetic and type 2 diabetic men.

Variable	Prediabetic men		Type 2 diabetic men		*p* value		
	pre (n = 5)	post (n = 4)	pre (n = 11)	post (n = 9)	Baseline difference	Time	Time × DM
[b]Fasting glucose (mmol/l)	6.6 (5.9, 7.5)	6.8 (6.0, 7.8)	7.5 (6.9, 8.1)	7.3 (6.7, 8.0)	0.12	0.86	0.40
[b]Fasting insulin (pmol/l)	12.0 (6.7, 21.5)	11.7 (6.4, 21.5)	15.8 (10.7, 23.5)	14.9 (9.9, 22.4)	0.43	0.73	0.88
Fasting NEFA (mmol/l)	0.82 (0.68, 0.97)	0.78 (0.62, 0.93)	0.63 (0.53, 0.73)	0.64 (0.53, 0.75)	0.033*	0.65	0.51
OGTT 2h glucose (mmol/l)	9.6 (6.7, 12.5)	9.0 (5.9, 12)	11.9 (9.9, 13.8)	10.9 (8.8, 12.9)	0.21	0.22	0.81
Pancreatic metabolism							
[a]Pancreatic fat (%)	8.7 (4.7, 13.8)	6.8 (3.3, 11.4)	8.7 (5.7, 12.3)	6.7 (4.1, 9.9)	0.86	0.14	0.97
Glucose uptake (μmol 100 g^{-1} min^{-1})	3.2 (2.6, 3.8)	3.7 (3.1, 4.3)	4.0 (3.6, 4.4)	3.9 (3.5, 4.4)	0.039*	0.11	0.032*
[a]Fatty acid uptake (μmol 100 g^{-1} min^{-1})	1.2 (0.9, 1.6)	1.3 (1, 1.7)	1.3 (1, 1.5)	1.2 (0.9, 1.5)	0.90	0.99	0.54
Beta-cell function							
ISR_{basal} (pmol min^{-1} m^{-2})	125 (84, 166)	114 (72, 157)	164 (137, 192)	151 (122, 179)	0.12	0.16	0.90
[b]ISR_{early} (nmol m^{-2})	9.1 (7.2, 11.5)	9.6 (7.5, 12.3)	9.0 (7.7, 10.6)	8.1 (6.8, 9.6)	0.97	0.65	0.21
[b]$\Delta ISR_{0-30}/\Delta G_{0-30}$ (nmol m^{-2}/mmol l^{-1})	0.11 (0.07, 0.18)	0.08 (0.05, 0.13)	0.07 (0.05, 0.09)	0.06 (0.04, 0.08)	0.060	0.030*	0.14
ISR_{late} (nmol m^{-2})	48 (39, 56)	42 (33, 52)	38 (32, 44)	41 (35, 47)	0.067	0.67	0.17
ISR_{total} (nmol m^{-2})	57 (48, 66)	52 (42, 62)	47 (41, 54)	49 (42, 56)	0.083	0.64	0.30
Glucose sensitivity (pmol min^{-1} $m^{-2}$$[mmol/l]^{-1}$)	88 (60, 115)	69 (38, 99)	48 (30, 67)	52 (32, 73)	0.017*	0.43	0.24
Rate sensitivity (pmol m^{-2} $[mmol/l]^{-1}$)	1059 (739, 1378)	710 (349, 1072)	575 (360, 791)	363 (122, 603)	0.052	0.047*	0.60
[a]Potentiation factor ratio	1.2 (0.6, 2.0)	1.9 (1.1, 2.9)	1.4 (1.0, 1.9)	1.6 (1.1, 2.2)	0.58	0.049*	0.30

The results are presented as model-based means (95% CI). Baseline difference *p* value indicates whether there is a baseline difference between prediabetic and type 2 diabetic men, Time *p* value displays the mean change between pre- and post-measurements, and Time × DM *p* value indicates whether the mean changes are different between prediabetic and type 2 diabetic men. ISR, insulin secretion rate; $\Delta ISR_{0-30}/\Delta G_{0-30}$, the ratio between the increment in ISR above baseline and increment in plasma glucose concentration above the fasting level at 30 min; [a]Square root transformation performed; [b]Logarithmic transformation (log_{10}) performed. *Statistically significant *p* value ($p \leq 0.05$).

ESM Table 2. Effects of exercise training on glycemic control, pancreatic metabolism and beta-cell function in prediabetic or type 2 diabetic men and women.

Variable	Prediabetic or type 2 diabetic men		prediabetic or type 2 diabetic women		*p* value		
	pre (n = 16)	post (n = 13)	pre (n = 10)	post (n = 8)	Baseline difference	Time	Time × sex
[b] Fasting glucose (mmol/l)	7.2 (6.8, 7.7)	7.2 (6.7, 7.7)	6.8 (6.3, 7.3)	6.8 (6.3, 7.4)	0.23	0.88	0.71
[b]Fasting insulin (pmol/l)	14.5 (10.9, 19.3)	13.9 (10.3, 18.7)	11.0 (7.6, 15.9)	12.2 (8.3, 17.7)	0.24	0.71	0.33
Fasting NEFA (mmol/l)	0.69 (0.61, 0.77)	0.68 (0.60, 0.77)	0.97 (0.86, 1.07)	0.91 (0.79, 1.04)	< 0.001*	0.33	0.47
OGTT 2h glucose (mmol/l)	11.2 (9.7, 12.6)	10.3 (8.8, 11.8)	10.3 (8.5, 12.1)	9.7 (7.8, 11.6)	0.45	0.11	0.75
Pancreatic metabolism							
[a]Pancreatic fat (%)	8.7 (6.2, 11.5)	6.7 (4.6, 9.2)	7.7 (5.0, 11.1)	7.1 (4.1, 10.8)	0.73	0.28	0.59
Glucose uptake (µmol 100 g^{-1} min^{-1})	3.7 (3.3, 4.1)	3.8 (3.4, 4.2)	4.3 (3.8, 4.7)	3.9 (3.4, 4.4)	0.092	0.30	0.055
[a]Fatty acid uptake (µmol 100 g^{-1} min^{-1})	1.3 (1.1, 1.4)	1.2 (1.0, 1.4)	1.9 (1.6, 2.2)	2.0 (1.7, 2.4)	0.001*	0.57	0.41
Beta-cell function							
ISR_{basal} (pmol min^{-1} m^{-2})	152 (132, 172)	140 (119, 160)	120 (95, 146)	116 (90, 143)	0.052	0.13	0.40
[b]ISR_{early} (nmol m^{-2})	9.1 (8.0, 10.3)	8.5 (7.5, 9.8)	8.7 (7.4, 10.3)	7.7 (6.4, 9.1)	0.70	0.037*	0.41
[b]$\Delta ISR_{0-30}/\Delta G_{0-30}$ (nmol m^{-2}/mmol l^{-1})	0.08 (0.06, 0.1)	0.07 (0.05, 0.08)	0.07 (0.05, 0.1)	0.06 (0.04, 0.08)	0.70	0.005*	0.52
ISR_{late} (nmol m^{-2})	41 (37, 45)	41 (36, 46)	41 (35, 46)	42 (36, 48)	0.97	0.73	0.86
ISR_{total} (nmol m^{-2})	50 (45, 55)	50 (45, 55)	50 (44, 56)	50 (43, 57)	0.89	0.96	0.96
Glucose sensitivity (pmol min^{-1} $m^{-2}[mmol/l]^{-1}$)	61 (46, 75)	57 (42, 73)	54 (36, 72)	52 (33, 71)	0.56	0.67	0.91
Rate sensitivity (pmol m^{-2} $[mmol/l]^{-1}$)	726 (529, 924)	459 (242, 676)	860 (609, 1110)	561 (284, 837)	0.46	0.007*	0.87
[a]Potentiation factor ratio	1.3 (0.9, 1.8)	1.7 (1.2, 2.3)	1.8 (1.3, 2.6)	2.4 (1.7, 3.3)	0.11	0.027*	0.79

The results are presented as model-based means (95% CI). Baseline difference *p* value indicates whether there is a baseline difference between men and women, Time *p* value displays the mean change between pre- and post-measurements, and Time × sex *p* value indicates whether the mean changes are different between men and women. ISR, insulin secretion rate; $\Delta ISR_{0\text{-}30}/\Delta G_{0\text{-}30}$, the ratio between the increment in ISR above baseline and increment in plasma glucose concentration above the fasting level at 30 min; [a]Square root transformation performed; [b]Logarithmic transformation ($\log_{10}$) performed. *Statistically significant *p* value ($p \leq 0.05$).

ESM Table 3. Participant characteristics and glycemic control in prediabetic or type 2 diabetic participants including men (n=16) and women (n=10) before and after SIT and MICT.

Variable	Prediabetic/T2DM SIT		Prediabetic/T2DM MICT		*p* value		
	pre (n = 13)	post (n = 11)	pre (n = 13)	post (n = 10)	Baseline difference	Time	Time × Group
men/women (n)	9/4	7/4	7/6	6/4			
prediabetic/T2DM participants (n)	2/11	1/10	7/6	6/4			
Age (years)	49 (47, 51)	-	49 (46, 51)	-	0.85		
Weight (kg)	91.6 (84.0, 99.2)	91.2 (83.6, 98.8)	92.0 (84.4, 99.6)	91.5 (83.9, 99.1)	0.95	0.086	0.96
BMI (kg/m^2)	29.9 (28.3, 31.5)	29.8 (28.2, 31.4)	31.0 (29.4, 32.6)	30.8 (29.2, 32.4)	0.36	0.075	0.84
Waist circumference (cm)	103.7 (98.5, 108.8)	103.5 (98.3, 108.6)	104.6 (99.4, 109.8)	103.7 (98.5, 108.9)	0.87	0.068	0.24
Fat (%)[b]	32.2 (28.1, 36.9)	31.2 (27.2, 35.8)	33.0 (28.8, 37.8)	32.0 (27.9, 36.8)	0.78	0.011*	0.92
Subcutaneous fat (kg)[a]	7.1 (5.7, 8.8)	6.9 (5.5, 8.6)	7.1 (5.7, 8.7)	6.9 (5.6, 8.6)	0.97	0.046*	0.90
Visceral fat (kg)[a]	3.1 (2.3, 4.1)	3.0 (2.2, 3.9)	3.6 (2.7, 4.7)	3.4 (2.6, 4.4)	0.46	0.007*	0.43
$\dot{V}O_{2peak}$ (ml kg^{-1} min^{-1})	27.0 (24.4, 29.7)	28.4 (25.7, 31.1)	27.4 (24.7, 30.1)	27.2 (24.5, 29.9)	0.83	0.14	0.048*
M value (µmol kg^{-1} min^{-1})	19.6 (13.0, 26.2)	24.8 (17.9, 31.6)	17.4 (11.3, 23.5)	19.3 (12.9, 25.7)	0.37	0.041*	0.32
HbA_{1c} (mmol/mol)	39.6 (36.8, 42.3)	37.8 (35.0, 40.6)	39.5 (36.8, 42.3)	37.5 (34.7, 40.3)	0.92	0.001*	0.79
HbA_{1c} (%)	5.8 (5.5, 6.0)	5.6 (5.4, 5.9)	5.8 (5.5, 6.0)	5.6 (5.3, 5.8)	0.92	0.002*	0.82
Fasting glucose (mmol/l)	7.4 (6.8, 7.9)	7.3 (6.7, 7.8)	6.8 (6.3, 7.3)	6.9 (6.4, 7.5)	0.22	0.94	0.42
Fasting insulin (pmol/l)[b]	12.8 (9.3, 17.8)	12.9 (9.2, 18.0)	13.3 (9.5, 18.4)	13.5 (9.6, 18.9)	0.86	0.88	0.93
Fasting NEFA (mmol/l)	0.74 (0.62, 0.85)	0.75 (0.63, 0.88)	0.84 (0.73, 0.95)	0.78 (0.66, 0.90)	0.43	0.43	0.17
OGTT 2h glucose (mmol/l)	11.2 (9.6, 12.8)	9.9 (8.3, 11.6)	10.4 (8.8, 12.1)	10.2 (8.5, 11.9)	0.82	0.092	0.23
OGTT 2h insulin (pmol/l)[b]	68.7 (49.0, 96.3)	64.6 (45.0, 92.7)	68.6 (49.0, 96.2)	63.7 (43.8, 92.8)	0.97	0.61	0.96
OGTT glucose AUC (mmol/l × min)	1336 (1218, 1453)	1305 (1182, 1427)	1295 (1177, 1413)	1305 (1179, 1431)	0.80	0.77	0.57

The result is presented as mean (95% CI) for age. For all other variables, the results are presented as model-based means (95% CI). Baseline difference *p* value indicates whether there is a baseline difference between SIT and MICT groups, Time *p* value displays the mean change between pre- and post-measurements, and Time × Group *p* value indicates whether the mean changes are different between SIT and MICT. T2DM, type 2 diabetes mellitus; SIT, sprint interval training; MICT, moderate-intensity continuous training; [a]Square root transformation performed; [b]Logarithmic transformation (log_{10}) performed. *Statistically significant *p* value ($p \leq 0.05$).

ESM Table 4. Pancreatic metabolism and beta-cell function in prediabetic or type 2 diabetic participants including men (n = 16) and women (n = 10) before and after SIT and MICT.

Variable	Prediabetic/T2DM SIT		Prediabetic/T2DM MICT		*p* value		
	pre (n = 13)	post (n = 11)	pre (n = 13)	post (n = 10)	Baseline difference	Time	Time × Group
Pancreatic fat (%)	8.5 (5.6, 11.3)	8.0 (5.0, 11.0)	9.3 (6.4, 12.1)	6.6 (3.6, 9.6)	0.73	0.21	0.37
Pancreatic metabolism							
Glucose uptake (μmol 100 g^{-1} min^{-1})	4.0 (3.6, 4.5)	3.8 (3.3, 4.3)	3.9 (3.5, 4.3)	4.0 (3.5, 4.4)	0.72	0.52	0.25
[a]Fatty acid uptake (μmol 100 g^{-1} min^{-1})	1.4 (1.1, 1.7)	1.5 (1.2, 1.8)	1.6 (1.3, 1.8)	1.5 (1.2, 1.8)	0.25	0.76	0.42
Beta-cell function							
ISR_{basal} (pmol min^{-1} m^{-2})	142 (119, 166)	136 (112, 160)	137 (114, 161)	125 (101, 150)	0.78	0.082	0.54
[b]ISR_{early} (nmol m^{-2})	8.5 (7.4, 9.8)	7.5 (6.5, 8.7)	9.4 (8.2, 10.8)	9.0 (7.8, 10.5)	0.41	0.056	0.33
[b]$\Delta ISR_{0-30}/\Delta G_{0-30}$, (nmol m^{-2}/mmol l^{-1})	0.075 (0.057, 0.099)	0.063 (0.047, 0.083)	0.075 (0.056, 0.100)	0.061 (0.045, 0.082)	0.97	0.005*	0.87
ISR_{late} (nmol m^{-2})	40 (35, 45)	42 (36, 47)	42 (37, 47)	41 (36, 47)	0.48	0.77	0.44
ISR_{total} (nmol m^{-2})	49 (43, 54)	49 (44, 55)	52 (46, 57)	50 (44, 56)	0.39	0.94	0.58
Glucose sensitivity (pmol min^{-1} $m^{-2}$$[mmol/l]^{-1}$)	57 (42, 73)	56 (39, 72)	59 (43, 74)	55 (38, 72)	0.91	0.64	0.88
Rate sensitivity (pmol m^{-2} $[mmol/l]^{-1}$)	706 (495, 918)	363 (134, 592)	849 (637, 1061)	647 (407, 887)	0.42	0.007*	0.44
[a]Potentiation factor ratio	1.6 (1.1, 2.2)	2.1 (1.5, 2.9)	1.5 (1.0, 2.0)	1.8 (1.2, 2.4)	0.75	0.030*	0.50

The results are presented as model-based means (95% CI). Baseline difference *p* value indicates whether there is a baseline difference between SIT and MICT groups, Time *p* value displays the mean change between pre- and post-measurements, and Time × Group *p* value indicates whether the mean changes are different between SIT and MICT. T2DM, type 2 diabetes mellitus; SIT, sprint interval training; MICT, moderate-intensity continuous training; ISR, insulin secretion rate; $\Delta ISR_{0-30}/\Delta G_{0-30}$, the ratio between the increment in ISR above baseline and increment in plasma glucose concentration above the fasting level at 30 min; [a]Square root transformation performed; [b]Logarithmic transformation (log_{10}) performed. *Statistically significant *p* value ($p \leq 0.05$).

ESM Table 5. Correlations between pancreatic fat content and whole-body and beta-cell parameters in prediabetic or type 2 diabetic participants including men (n = 16) and women (n = 10).

Variable	Pancreatic fat content (%)			
	baseline, prediabetic/T2DM participants		changes, prediabetic/T2DM participants	
	r	*p*	*r*	*p*
BMI (kg/m^2)	-0.09	0.70	-0.06	0.83
Fat (%)	-0.15	0.54	-0.06	0.83
Visceral fat (kg)	-0.12	0.60	-0.02	0.94
M value (μmol kg^{-1} min^{-1})	0.16	0.52	-0.31	0.27
HbA_{1c} (mmol/mol)	-0.02	0.92	-0.15	0.60
Fasting glucose (mmol/l)	-0.29	0.21	-0.36	0.19
Fasting insulin (pmol/l)	-0.05	0.83	-0.41	0.13
Fasting NEFA (mmol/l)	-0.06	0.81	0.20	0.53
Pancreatic glucose uptake (μmol 100 g^{-1} min^{-1})	-0.11	0.65	-0.44	0.12
Pancreatic fatty acid uptake (μmol 100g^{-1} min^{-1})	-0.17	0.51	0.17	0.60
ISR_{basal} (pmol min^{-1} m^{-2})	-0.05	0.82	-0.18	0.53
ISR_{early} (nmol m^{-2})	0.11	0.64	-0.56	0.032*
ISR_{total} (nmol m^{-2})	0.11	0.64	-0.58	0.027*
Glucose sensitivity (pmol min^{-1} m^{-2} [mmol/l]$^{-1}$)	-0.01	0.98	0.13	0.65
Rate sensitivity (pmol m^{-2} [mmol/l]$^{-1}$)	-0.08	0.74	-0.29	0.30
Potentiation factor ratio	-0.35	0.13	-0.03	0.92

T2DM; type 2 diabetes mellitus; ISR, insulin secretion rate. *Statistically significant *p* value ($p \leq 0.05$).
